# Medical Items

**Published:** 1908-06

**Authors:** 


### MEDICAL ITEMS

CYSTITIS.—It should not be forgotten how prominently a condition of hyperacidity of the urine figures as an etiological factor in the ordinary case of acute cystitis. Proof of this is found in the readiness with which such cases yield to Alkalithia. This is the alkaline treatment in a form which permits of the alkalies being pushed to the point of alkalinizing the secretions without disturbing the stomach as with the use of the plain alkalies.

### COMPARATIVE POTENCY OF HYOSCINE AND SCOPOLAMINE HYDROBROMIDE IN REFRACTION WORK: EVIDENCE AS TO UNMISTAKABLE NON-INDENTITY.

Dr. Wendell Reber, of Philadelphia contributes an interesting article upon this subject in The Journal of the American Medical Association for April 25. His paper was read in the Section on Ophthalmology of the American Medical Association at its Atlantic City meeting in June, 1907. For some unexplainable reason this article, which bears so strongly upon the controversy concerning the alleged identity of hyoscine and scopolamine, has been withheld from publication for eleven months. This is of peculiar interest, inasmuch as the editor of the Association Journal during this period has been asserting and reasserting most vociferously through the columns of that jouranl that these two alkaloids are both chemically and pharmacodynamically indentical. Dr. Reber's conclusions, which were based upon careful experimental work made upon human beings, are diametrically opposed to the assertions of Dr. C. H. Simmons, Dr. H. C. Wood, Jr., and others, in The Journal of the American Medical Association and its "anvil chorus."

Dr. Reber was led to these experiments by an experience reported in The American Journal of Pharmacy in 1899. At that time he found that when one drop of a 1-10 per cent. solution of hyoscine hydrobromide was used in the right eye, and one drop

